# A Simple, Rapid, and Contamination-Free Ultra-Sensitive *Cronobacter sakazakii* Visual Diagnostic Platform Based on RPA Combined with CRISPR/Cas12a

**DOI:** 10.3390/foods14173120

**Published:** 2025-09-06

**Authors:** Yan Liu, Yu Xie, Zhangli Wang, Zuoqi Gai, Xu Zhang, Jiahong Chen, Hongtao Lei, Zhenlin Xu, Xing Shen

**Affiliations:** 1 Guangdong Provincial Key Laboratory of Food Quality and Safety, College of Food Science, South China Agricultural University, Guangzhou 510642, China; ly191107@163.com (Y.L.); xy2356892021@163.com (Y.X.); 15108651257@163.com (Z.W.); jiahongchen@scau.edu.cn (J.C.); hongtao@scau.edu.cn (H.L.); jallent@163.com (Z.X.); 2 School of Agricultural and Biological Engineering, Foshan University, Foshan 528225, China; gaizuoqi@126.com (Z.G.); Fuzhangxu@fosu.edu.cn (X.Z.)

**Keywords:** *Cronobacter sakazakii*, rapid detection, CRISPR/Cas12a, recombinase polymerase amplification, one-pot method

## Abstract

CRISPR/Cas systems have made significant progress in the field of molecular diagnostics in recent years. To overcome the aerosol contamination problem brought on by amplicon transfer in the common two-step procedure, the “one-pot method” has become a major research hotspot in this field. However, these methods usually rely on specially designed devices or additional chemical modifications. In this study, a novel “one-pot” strategy was developed to detect the foodborne pathogen *Cronobacter sakazakii* (*C. sakazakii*). A specific sequence was screened out from the virulence gene *ompA* of *C. sakazakii* as the detection target. Combining with the recombinase polymerase amplification (RPA), a rapid detection platform for *C. sakazakii* based on the CRISPR/Cas12a system was established for the first time. The sensitivity of this method was determined from three different levels, which are 10^−4^ ng/μL for genomic DNA (gDNA), 1.43 copies/μL for target DNA, and 6 CFU/mL for pure bacterial culture. Without any microbial enrichment, the detection limits for artificially contaminated cow and goat milk powder samples were 4.65 CFU/mL and 4.35 CFU/mL, respectively. To address the problem brought on by aerosol contamination in the common RPA-CRISPR/Cas12a two-step method, a novel pipette tip-in-tube (PTIT) method for simple and sensitive one-pot nucleic acid detection was further developed under the inspiration of the capillary principle. The RPA and CRISPR/Cas systems were isolated from each other by the force balance of the solution in a pipette tip before amplification. The detection limits of the PTIT method in pure bacterial culture and the spiked samples were exactly the same as that of the two-step method, but with no false positive cases caused by aerosol contamination at all. Compared with other existing one-pot methods, the PTIT method requires no additional or specially designed devices, or any chemical modifications on crRNA and nucleic acid probes. Therefore, the PTIT method developed in this study provides a novel strategy for realizing one-pot CRISPR/Cas detection easily and holds significant potential for the rapid point-on-care testing (POCT) application.

## 1. Introduction

The clustered regularly interspaced short palindromic repeats (CRISPR) with their associated protein (Cas) systems have become a novel biological tool in the last decade, with functions such as gene editing and transcriptional regulation [1]. They have also made significant advancements in the field of molecular diagnostics in recent years [2,3]. Cas proteins, the most commonly used being Cas12a or Cas13a for diagnostics, can specifically cleave the target nucleic acid sequence under the guidance of crRNA [4,5]. Subsequently, the trans-cleavage of Cas12a is triggered, resulting in the indiscriminate cleavage of single-stranded DNA (ssDNA) near the target nucleic acid, thereby enabling signal generation for detection [4]. To enhance the efficiency of the detection, nucleic acid amplification is usually employed before Cas cleavage. The RPA and CRISPR/Cas have been developed as a classic mode for nucleic acid detection so far [4,6]. Although this two-step method is highly sensitive and capable of detecting single-copy genes, it can easily lead to aerosol contamination during the transfer of amplificons and can result in false-positive outcomes [7]. To address this issue, one-pot methods that contain both the RPA and CRISPR systems in one tube have become a research hotspot [8]. The one-pot methods avoid the operation of opening the tube during the reaction, thereby preventing aerosol contamination, making them suitable for POCT in complex environmental conditions.

The earliest one-pot method directly mixed RPA and CRISPR/Cas system together, achieving the purpose of rapidly detecting the SARS-CoV-2 in a closed tube. However, the sensitivity of this method was much lower than the two-step method [9]. In this mixed system, amplification and cleavage happen at the same time. This makes it hard to accumulate enough amplicons before the Cas proteins’ trans-cleavage activity is activated, leading to non-specific degradation of amplification primers and a sharp drop in reaction efficiency [4,7,10]. Later researchers have been dedicated to developing all kinds of strategies to realize one-pot detection. These strategies mainly fall into two classes: cleavage control and physical isolation.

Cleavage control is aimed at delaying or reducing the cleavage activity of Cas protein, for example, conducting experiments with suboptimal crRNA [11,12,13], modifying or blocking the crRNA, etc. [14,15,16]. These cleavage control strategies have no need to employ additional devices and processing steps. However, they usually require the introduction of additional additives or external activation, as well as complex and expensive crRNA modifications, and thus cannot be widely applied yet. Physical isolation mainly separates the RPA system from the CRISPR system until the amplification is completed. For example, special devices have been designed to separate the two systems into inner and outer tubes [17,18,19], or additives like glycerol are added to achieve phase separation [7,20,21]. Physical isolation strategies can maintain the high sensitivity of the two-step method, but they often rely on specially designed devices, additional centrifugation operations, or unstable phase isolation systems. These existing methods have demonstrated the realizability of the one-pot method, but more novel strategies are still pursued to meet the requirements of low-cost, simple, rapid, and ultra-sensitive on-site detection.

*C. sakazakii* is a foodborne pathogen that resides in the intestines of humans and animals [22]. It is a facultative anaerobic, Gram-negative bacterium of the genus *Cronobacter* (*Enterobacteriaceae* family), which also comprises six other species including *C. malonaticus* and *C. turicensis* [23]. Among all species, *C. sakazakii* is the main pathogen associated with human diseases at a low infectious dose [24]. It has been isolated from a variety of foods, including dairy products, dry foods, cereals, spices, and meats, especially powdered infant formula (PIF) [25,26]. One of the main reasons why *C. sakazakii* can contaminate PIF is that it is highly resistant to drying out, thus retaining high activity even after more than two years [27,28]. *C. sakazakii* can invade the epithelial cells of infants’ small intestines and survive in macrophages. It spreads throughout the body via the bloodstream, crosses the blood–brain barrier, and causes severe diseases—such as bacteremia, meningitis, and necrotizing enterocolitis—with a high mortality rate of around 80% [29,30]. Therefore, countries all around the world, like the European Union, the USA, and China, all stipulate that *Cronobacter* species must not be detected in PIF. However, incidents caused by *Cronobacter* contamination, in most cases by *C. sakazakii*, have still been reported in recent years and have resulted in dozens of deaths of infants and young children [31]. Under this situation, developing rapid and sensitive detection methods of *C. sakazakii* is a crucial way of preventing and controlling such safety issues.

In this study, a highly convenient spatiotemporal separation-based CRISPR/Cas analysis system—pipette tip-in-tube (PTIT)—was developed to realize low-cost one-pot detection of *C. sakazakii*. A fragment on the virulence gene *ompA* of *C. sakazakii* was screened out as a detection target. The RPA and CRISPR/Cas systems were isolated just by a pipette tip under the effect of capillary force, and mixed by simply shaking to produce the fluorescence signal. Compared with previous one-pot methods, the PTIT method did not require special devices, any additives, or modified crRNAs, but provided super sensitive results in a short time. This study established a novel method for the rapid detection of *C. sakazakii*, and also provided a new way to achieve on-site detection using the CRISPR system either in the laboratory or in other environments.

## 2. Materials and Methods

### 2.1. Materials and Reagents

*C. sakazakii* (CICC 21560), *C. malonaticus* (CICC 21551), and *C. turicensis* (CICC 24178) were purchased from the China Center of Industrial Culture Collection (CICC). *Escherichia coli* (*E. coli*), *Staphylococcus aureus* (*S. aureus*), *Bacillus citreus* (*B. citreus*), *Salmonella*, *Listeria monocytogenes* (L. *monocytogenes*), *Bacillus cereus* (*B. cereus*), and *Vibrio parahaemolyticus* (*V. parahaemolyticus*) were preserved in our lab (Guangdong Provincial Key Laboratory of Food Quality and Safety). Real-Time PCR Detection Kit for *C. sakazakii*, nutrient broth (NB), luria-Bertani (LB), and agar powder medium (NA) were procured from Huankai Microbial Sci. & Tec. Co., Ltd. (Guangzhou, China). DNA oligonucleotides and the CRISPR RNA were chemically synthesized by Genewiz Biotechnology (Suzhou, China). The TIANamp Bacteria DNA Kit and TIANquick Midi Purification Kit were purchased from Tiangen Biochemical Technology (Beijing, China). The TwistAMP DNA amplification kit was bought from TwistDX Ltd. (Cambridge, UK). The dNTPs (10 mM each) were purchased from TansGen Biotech Co., Ltd. (Beijing, China). The single-stranded DNA fluorescent reporter probe (ssDNA-FQ) (FAM-TTTTTT-BHQ) and Cas12a enzyme were obtained by Editgene Co., Ltd. (Guangzhou, China). The RNase inhibitor was prepared by Xinhai Gene Testing (Harbin, China). NEBuffer2.1 was purchased from New England Biolabs Inc. (Ipswich, MA, USA). The infant cow milk powder was acquired from Yili company (Hohhot, China), and the infant goat milk powder was purchased from BeiKangXi company (Changsha, China).

### 2.2. Bacterial Strain Cultivation and Genomic DNA Extraction

A total of 10 μL each of *C. sakazakii* and *C. malonaticus*, preserved in glycerol, was added to the 5 mL NB liquid medium. For other bacterial strains, 10 μL of each was put into the 5 mL LB liquid medium. All cultures were incubated at 37 °C and 250 rpm overnight [32,33]. High-purity gDNA was extracted using the TIANamp Bacteria DNA Kit according to the manufacturer’s instructions. To simplify the extraction steps as well as to evaluate the effect of the complex matrix of milk powder on the gDNA extraction, this study also employed a simple boiling method to extract the gDNA of *C. sakazakii* from artificially contaminated PIF directly [34,35]. The extraction process was as follows: 1 mL of the artificially contaminated milk powder solution was taken and centrifuged at 12,000 rpm for 2 min, and the supernatant was discarded. Then the pellet was washed twice with 200 μL of ultrapure water (ddH_2_O). Afterward, the pellet was dissolved with 100 μL of ddH_2_O and boiled in a water bath at 100 °C for 10 min. Finally, the solution was centrifuged at 12,000 rpm for 2 min; afterwards, the tube was placed on ice for 2 min, and the supernatant was collected. The concentration and purity of the extracted gDNA was quantified using Nanodrop 2000 (Thermo Fisher Scientific Inc., Waltham, MA, USA), and stored at −20 °C for future experiments.

### 2.3. Design and Screening for RPA Primers and crRNA

After the screening of more than ten feature gene fragments of *C. sakazakii*, the virulence gene *ompA* was finally chosen as the detection target. The full sequence of the *ompA* gene (GQ 845410.1) was downloaded from the NCBI database (https://www.ncbi.nlm.nih.gov/) (accessed on 15 July 2023). Nearly 5000 homologous sequences of the *ompA* gene were retrieved via BLAST in NCBI, and were downloaded for sequence alignment using SnapGene software 4.2.4. First, the PAM site was located, and a specific 21 bp fragment on *ompA* was selected as the target. After that, the crRNA was designed based on the target. Six pairs of primers were designed by Primer Premier 5 and SnapGene based on the chosen target (Table S1), according to the TwistDx^®^ Assay Design Manual. The secondary structure and heterodimer levels of primers were analyzed using the IDT Oligo Analyzer website (https://sg.idtdna.com/calc/analyzer) (accessed on 22 July 2023). The gDNA of *C. sakazakii* was used as a template for the RPA reaction system to evaluate the amplification efficiency of these primers. The amplicons were purified and analyzed with agarose gel electrophoresis to obtain the primer pairs that produced a single band with the correct length. Subsequently, the gDNA of *E. coli*, *S. aureus*, *Salmonella*, *B. cereus*, L. *monocytogenes*, *V. parahaemolyticus*, and *B. citreus* were used as templates for the RPA reaction to verify the specificity of the optimal primer pair. The sequences of all final primers and probe are shown in Table S2.

### 2.4. Two-Step RPA-CRISPR/Cas12a System Construction

A two-step system was first established to explore the optimal reaction parameters of the RPA and CRISPR/Cas12a system, using the gDNA of *C. sakazakii* as template. To achieve optimal detection performance, the effects of primer concentration, dNTP concentration, amplification temperature, and amplification time on the amplification efficiency were evaluated. In order to reduce testing costs, the volume of the RPA reaction system was reduced to 10 μL. The primer concentrations were set from 0.24 μM to 0.64 μM, while the concentrations of dNTPs were selected from 0.8 mM to 2.8 mM. Meanwhile, the reaction temperatures were tested from 36 °C to 41 °C, and the reaction time ranged from 10 min to 35 min to meet the requirements for rapid detection.

At the same time, the concentrations of several key components in the CRISPR/Cas12a system were optimized. The volume of the CRISPR/Cas system was set at 25 μL. Firstly, the Cas12a enzyme concentrations were diluted to 40 nM, 60 nM, 80 nM, 100 nM, 120 nM, and 140 nM to determine the most suitable Cas12a concentration. Then, the concentrations of crRNA and ssDNA-FQ were optimized. The ratios of crRNA to Cas12a were chosen as 0:1, 0.5:1, 1:1, 1.5:1, 2:1, 2.5:1, and 3:1, while the ratios of ssDNA-FQ to Cas12a were set as 0:1, 0.5:1, 1:1, 1.5:1, 2:1, 2.5:1, and 3:1 for the experiments. After individual amplification, the entire RPA system was added to the prepared CRISPR system, then the mixed solution was incubated in a water bath at 37 °C for 10 min. The fluorescence can be observed by the naked eye using a mini BluView transilluminator (Eastwin Life Sciences, Inc., Beijing, China) with a wavelength of 470 nm, and fluorescence values were also measured by a QuantStudio 3 Real-Time PCR System (Thermo Fisher Scientific Inc., Waltham, MA, USA).

### 2.5. Preliminary Test of the PTIT (Pipette Tip-in-Tube) Method

The idea of the PTIT method was inspired by the capillary principle, and pigment solution was used to pre-verify that the system would perform controlled separation and mixing as expected before the method was established. A sufficiently concentrated carmine solution was prepared to replace the RPA system. Following the procedure described in the last paragraph, a pipette tip containing 10 μL of carmine solution was placed into a centrifuge tube with 15 μL of ddH_2_O. After incubating at 37 °C for 30 min, the situation inside the tube was observed. If the carmine solution in the pipette tip did not flow out, the tube was shaken or centrifuged (several seconds) to observe whether any changes occurred within the tube.

### 2.6. The PTIT Method Procedure

In order to solve the aerosol contamination problem during liquid transfer of the two-step method, a highly convenient RPA-CRISPR/Cas12a one-pot method was developed in this study. The main operation procedure was as follows. Firstly, all RPA reaction solutions with template DNA and the CRISPR/Cas12a system were prepared in two tubes. Next, the RPA system was drawn up by a 10 μL pipette tip in a “draw twice, hit once” manner. The detailed operation procedure was pressing the plunger all the way down to the second stop to aspirate the liquid, inverting the pipette with the tip facing upward, and then removing the pipette tip. Then, the pipette tip carrying the RPA solution was placed vertically into the tube containing the CRISPR/Cas12a system. After a period of time, the tube was shaken by hand or centrifuged to allow the RPA reaction solution to come into the tube and contact with the CRISPR/Cas12a reaction system. Finally, the two systems were slowly blended in the tube and then incubated at 37 °C for another 20 min. Then, the fluorescence was observed as described above.

### 2.7. Establishment of the PTIT System

Based on the two-step system, the reaction system of the PTIT method was further optimized. The appropriate spatial mode of the two systems was first investigated. The RPA system was placed in the pipette tip, while the CRISPR/Cas12a system was placed in the outer tube, or their positions were exchanged to evaluate the detection effect in two ways. Next, to enhance the signal intensity, the ratios of ssDNA-FQ/Cas12a were set to 0:1, 1:1, 2:1, 3:1, 4:1, 5:1, and 6:1 to explore higher fluorescent probe concentrations. In the NTC experiment, the template was replaced with ddH_2_O, and the ssDNA/Cas12a ratio was set to 6:1 to find out the background fluorescence intensity under the highest concentration of ssDNA-FQ. In order to measure the fluorescence intensity precisely, the excess part of the pipette tip that exceeded the tube was cut off with scissors so that it could be placed into the PCR tube. The endpoint fluorescence values thus could be measured using the QuantStudio 3 Real-Time PCR System to make more accurate judgements.

### 2.8. Evaluation of Specificity and Sensitivity

In addition to three *Cronobacter* strains, *E. coli*, *S. aureus*, *Salmonella*, *B. cereus*, *B. citreus*, L. *monocytogenes*, and *V. parahaemolyticus* were also tested to evaluate the specificity of the RPA-CRISPR/Cas12a detection platform. The gDNA of the above bacteria was extracted and used as a template for the RPA reaction.

The sensitivity of the two-step RPA-CRISPR/Cas12a method was evaluated from three different levels: the concentration of gDNA, the concentration of pure bacterial culture, and the target DNA copies. First, the gDNA of *C. sakazakii* was diluted with a 10-fold gradient from 10^1^ ng/μL to 10^−5^ ng/μL and detected by this method to determine the detection limit at the gDNA level. Second, the *C. sakazakii* culture was diluted to different concentrations and counted according to the Chinese National Standard GB4789.40-2016 to obtain the number of colonies [36]. Afterwards, 1 mL of bacterial cultures from 10^6^ CFU/mL to 10^0^ CFU/mL was provided for DNA extraction and detection. Third, the RPA amplicons of the target sequence were recovered and diluted into a series of concentrations from 10^5^ copies/μL to 10^0^ copies/μL with ddH_2_O. These diluted target fragments were used as templates in the RPA reaction to measure the detection limit of target DNA.

As to the PTIT one-pot method, which was established on the basis of the two-step RPA-CRISPR/Cas12a platform, the same methods described above were used to assess the sensitivity of the PTIT method.

### 2.9. Sample Detection

Infant cow milk powder and infant goat milk powder products were purchased as real samples to verify the practicality of the established methods. Before testing, Real-Time PCR was utilized to confirm the absence of *C. sakazakii* contamination in the food samples according to the Industry Standard of the People’s Republic of China for Entry-Exit Inspection and Quarantine SN/T 1632.3-2013, Determination of *Enterobacter sakazakii* (*Cronobacter* spp.) from dehydrated powdered milk for export-Part 3: Real-Time PCR method for *Cronobacter* spp. [37]. Specifically, 10 g of cow milk powder or 10 g of goat milk powder was added to 90 mL of sterile physiological saline to prepare a 10-fold diluted sample solution. Subsequently, these solutions were aliquoted into tubes containing 4.5 mL each, with 500 μL of logarithmic-phase bacterial culture added to the first tube [35,38]. Serial 10-fold dilutions were performed to obtain artificially contaminated milk powder samples with final concentrations ranging from 10^6^ CFU/mL to 10^0^ CFU/mL. To assess the impact of the complex food matrix on different DNA extraction methods, 1 mL of each gradient-diluted spiked sample was taken for DNA extraction using both the commercial kit and the rapid boiling methods, respectively, and then tested by the two-step RPA-CRISPR/Cas12a and the PTIT methods.

### 2.10. Method Validation

To validate the accuracy of the established methods, a commercial *C. sakazakii* Real-Time PCR detection kit was employed for method validation. All DNA extracted from pure bacterial cultures with varying concentrations (10^3^ CFU/mL, 10^2^ CFU/mL, 10^1^ CFU/mL, and 10^0^ CFU/mL) using the kit-based extraction method were analyzed by Real-Time PCR and the two methods developed in this study at the same time. The results were compared with each other. A cycle threshold (Ct) value ≤ 35 was reported as a positive result for *C. sakazakii*. For Ct values between 35 and 37, the test was repeated. If the Ct value was ≥37, the result was considered negative for *C. sakazakii*; otherwise, it was reported as positive.

## 3. Results

### 3.1. Screening of RPA Primers and crRNA

**Figure 1:**
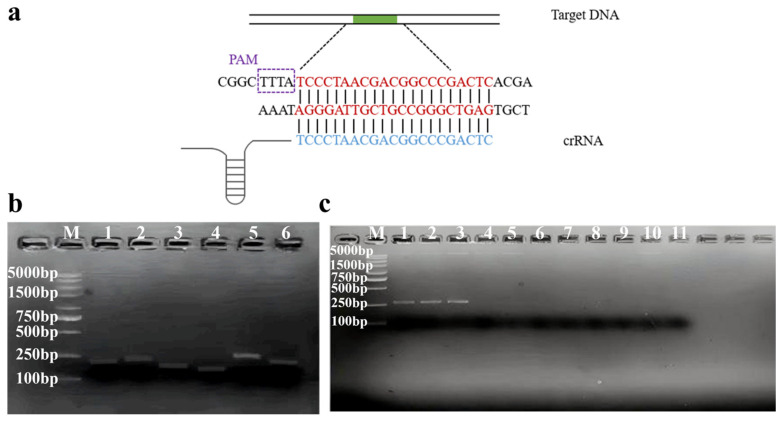
Screening of RPA primers and crRNA. (**a**) Schematic of dsDNA target detected with the Cas12a/crRNA. crRNA was provided in detail. The dsDNA target site was highlighted in red and crRNA fragments in blue. (**b**) Screening of RPA primers for *ompA* were visualized using 2% agarose gel electrophoresis. M: 5000 bp DNA marker; numbers from 1 to 6: F1R1, F2R2, F3R3, F4R4, F5R5, F6R6. (**c**) The specificity of RPA primer pair 5 for each enterotoxin gene was visualized using 2% agarose gel electrophoresis. M: 5000 bp DNA marker; numbers from 1 to 11: *C. sakazakii* (CICC 21560), *C. malonaticus* (CICC 21551), *C. turicensis* (CICC 24178), *E. coli*, *Salmonella*, *B. cereus*, L. *monocytogenes*, *S. aureus*, *V. parahaemolyticus*, *B. citreus*, no target control.

Several genes from *C. sakazakii* were screened, including *16S rRNA*, *23S rRNA*, *MMS*, *ompW*, and *ompA*. Ultimately, a specific 21 bp sequence that contains the PAM site was found in the virulence gene of *ompA*. Based on this sequence, the corresponding crRNA was designed. The specific fragment was highlighted in red in Figure 1a, while the complementary crRNA was indicated in blue.

According to the principle of RPA primer design, six pairs of primers were designed to amplify the target sequence containing this specific fragment, and the amplicons were analyzed using 2% agarose gel electrophoresis. The results showed that all six primer pairs successfully generated a single target band, and primer pair 5 (F5R5), which produced the brightest single band among them, was selected as the primer for RPA amplification (Figure 1b). Subsequently, the specificity of F5R5 was confirmed using gDNA from two other *Cronobacter* strains and various other foodborne pathogens as templates. As shown in Figure 1c, *C. malonaticus* and *C. turicensis* also produced the same targeted band as *C. sakazakii*, while no specific bands were generated by other foodborne pathogens or the blank control, indicating that F5R5 primers were specific for the *Cronobacter* genus.

### 3.2. Optimization of RPA Reaction

To obtain the best amplification performance, several key parameters were optimized. As the primer concentration increased, the brightness of bands gradually increased, but remained constant after reaching 0.56 μM (Figure S1a). Considering that a high primer concentration may lead to primer dimers, 0.56 μM was determined to be the optimal primer concentration. dNTPs provide the nucleotide substrates for the RPA reaction. However, too high a concentration of dNTPs can result in non-specific amplification. As shown in Figure S1b, the brightness of the bands increased slightly as the concentration of dNTPs increased. For the sake of saving cost, 2 mM was selected as the optimal concentration of dNTPs. For the reaction temperature, the results indicated that, except for a slightly lower brightness at 36 °C, the brightness of bands were similar between 37 °C and 41 °C (Figure S1c). Therefore, 37 °C was chosen as the reaction temperature for RPA to keep it consistent with the CRISPR/Cas12a system. Theoretically, within a certain range, the longer the amplification time lasts, the more the amplicons accumulate. The results showed that the bands became progressively brighter with increasing time (Figure S1d). To save detection time, the optimal reaction time was determined as 25 min. In summary, the reaction mixture of the RPA system consisted of 0.56 μM forward primer, 0.56 μM reverse primer, 2 mM dNTPs, 0.5 μL MgOAc, 3.98 μL C buffer, 1 μL L buffer, 2.4 μL P buffer, and 0.5 μL DNA template, with a reaction time of 25 min at 37 °C.

### 3.3. Establishment of the Two-Step RPA-CRISPR/Cas12a Detection Platform

The procedure of the two-step RPA-CRISPR/Cas12a system was as follows. The gDNA of the bacteria was added to the RPA system for amplification, which was carried out for 25 min. After amplification, the entire 10 μL of the amplicons was transferred to the CRISPR/Cas12a solution, the reaction continued for 10 min, and the fluorescence was observed or measured immediately. The entire process was conducted at 37 °C, and the test can be completed within 35 min.

**Figure 2:**
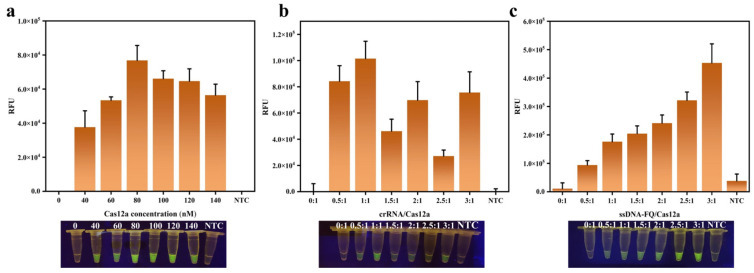
Establishment of the two-step RPA-CRISPR/Cas12a detection platform. (**a**) Fluorescence intensity produced at different Cas12a concentrations. (**b**) Fluorescence intensity generated from different crRNA/Cas12a ratios. (**c**) Fluorescence intensity generated from different ssDNA-FQ/Cas12a ratios. Error bars represent the mean ± standard deviation (SD) from three replicates. NTC represents no target control.

During the reaction, Cas12a enzyme is bound to crRNA first and recognizes the target DNA subsequently, forming a Cas12a-crRNA-DNA ternary complex. The cis-cleavage activity of Cas12a is activated by the complex, followed by the activation of its trans-cleavage activity, which leads to the random cleavage of nearby ssDNA-FQ [4,5]. In this reaction system, Cas12a, crRNA, and ssDNA-FQ are the key factors that decide the endpoint fluorescence intensity. It can be seen from Figure 2a that the fluorescence values first increased and then decreased as the Cas12a concentration increased. Therefore, the optimal concentration of 80 nM for Cas12a was chosen at the maximum fluorescence value. After the Cas12a concentration was determined, the ratio of crRNA/Cas12a was optimized. As shown in Figure 2b, the fluorescence values did not follow a specific law with varying crRNA/Cas12a ratios. However, the highest fluorescence intensity was observed when crRNA/Cas12a was 1:1. It is likely that the binding of crRNA to Cas12a reaches a best state at 1:1, whereas lower or higher concentrations of crRNA may affect the binding efficiency to Cas12a and thus affect its cleavage activity [39,40]. The ratio of ssDNA-FQ/Cas12a was investigated at the same time. The result indicated that the endpoint fluorescence intensity continued to increase as the ssDNA-FQ/Cas12a ratio increased (Figure 2c). Therefore, a highest ratio of 3:1 with tolerable fluorescence background was chosen as the best ssDNA-FQ/Cas12a ratio. Ultimately, the CRISPR/Cas12a system consisted of 80 nM of Cas12a, 80 nM of crRNA, 240 nM of ssDNA-FQ, 2.5 μL of 10 × NEBuffer 2.1, 0.5 μL of an RNase inhibitor (0.8 U/μL), and 10 μL of the amplicons, with ddH_2_O added to a final volume of 25 μL.

### 3.4. The Development of PTIT Method

**Figure 3:**
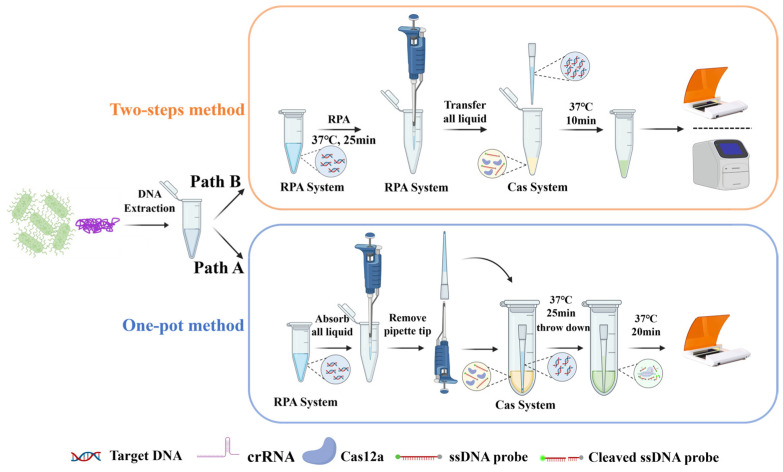
Schematic illustration of the traditional two-step method and the spatiotemporal separation-based CRISPR/Cas detection (RTIT) system. (Path A) The operating procedure of the PTIT method. (Path B) The operating procedure of the two-step method.

Based on the two-step RPA-CRISPR/Cas12a system, a novel one-pot method named PTIT (pipette tip-in-tube) was developed to eliminate the risk of aerosol contamination that usually happened in the two-step system during the amplicon transferring. The main operating procedure of the PTIT method is shown in Path A of Figure 3, while Path B represents the classic steps of the two-step method. Inspired by the capillary principle, the PTIT method utilized a common pipette tip to isolate the RPA system from the CRISPR/Cas reaction solution in a closed tube. The RPA system, including the gDNA, is first aspirated into a pipette tip following a “draw twice, hit once” manner, and a small air bubble is trapped at the tip of the pipette tip. Subsequently, the pipette tip is placed into a 1.5 mL Eppendorf tube containing the CRISPR/Cas reaction solution. In this situation, the force balance between the trapped bubble, the capillary force, and the gravity of the liquid was created, thus the liquid inside the pipette tip will not flow out of the tip and the two systems will remain isolated. After incubating at 37 °C for 25 min, the isolation status was broken by external forces such as shaking by hand or brief centrifugation at 10,000 rpm for several seconds. Then, the amplicons in the pipette tip will flow out and make contact with the CRISPR/Cas solution, which will diffuse into the pipette tip and mix with the RPA system under the capillary force. The tube continued to incubate at 37 °C for another 20 min, and the cleavage activity of Cas12a was activated to produce a fluorescent signal. This operation did not require additional physical isolation devices or costly chemical modifications, making it highly convenient.

**Figure 4:**
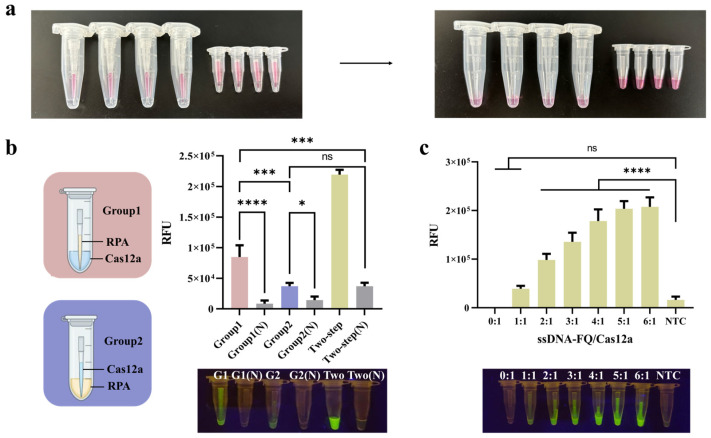
Establishment of the one-pot (PTIT) system. (**a**) Proof of concept of the PTIT method. (**b**) The endpoint fluorescence intensity of the RPA and CRISPR/Cas 12a system at two opposite positions. (**c**) The optimization of different ssDNA-FQ/Cas12a ratios. Error bars represent the mean ± standard deviation (SD) from three replicates. N represents no target control. Two-tailed Student’s *t*-test was used for each two-group comparison and one-way ANOVA test was used to compare all groups with no target control: * *p* < 0.05; *** *p* < 0.001; **** *p* < 0.0001; ns, not significant.

To test the feasibility of this idea, the PTIT method was first conducted with a high concentration of cochineal magenta solution instead of the RPA system. The results showed that, after 30 min or even longer, the carmine solution within the pipette tip did not leak out, and the ddH_2_O in the tube remained completely clear. After 30 min, the carmine solution was released from the pipette tip by shaking by hand, resulting in a uniform carmine-colored mixture in both the inner and outer tubes (Figure 4a). The results indicate that this method can effectively isolate the two solutions and allow for easy control of mixing.

### 3.5. The Optimization of the PTIT System

The PTIT method was established on the basis of the two-step system; therefore, only the relative position of the two systems and the ssDNA-FQ/Cas12a ratio needed to be adjusted. Using gDNA of *C. sakazakii* as the templates, two groups of experiments were performed by exchanging the positions of the two systems. As shown in Figure 4b, higher fluorescence intensity was observed when the RPA reaction occurred in the pipette tip rather than in the outer tube. Therefore, the PTIT mode was settled as the RPA system in the pipette tip, while the CRISPR/Cas system was in the outer tube. This could be attributed to the fact that the RPA system is more viscous, and it is easier to maintain the force balance when kept in the pipette tip and is less likely to flow down spontaneously. In contrast, during the RPA reaction, a portion of the Cas system may flow down, resulting in premature reactions and thus affecting the detection results.

As is known to all, a higher concentration of the ssDNA-FQ results in stronger fluorescence intensity for positive results, making it easier for visual observation. However, too high of a probe concentration can lead to a high background value and cause a false positive result. Therefore, we did not use a high concentration of ssDNA-FQ in the easily contaminated two-step method. However, in the PTIT one-pot method, we hope the results can be observed by the naked eye in POCT. Thus, the amount of ssDNA-FQ probes was promoted. The results (Figure 4c) showed that fluorescence intensity increased while the ssDNA-FQ/Cas12a ratio became higher, but the upward trend gradually slowed down. In addition, the fluorescence background was too high at the ratio of 6:1. Therefore, the ssDNA-FQ/Cas12a ratio was determined as 5:1.

### 3.6. Evaluation of the Assays’ Specificity and Sensitivity

**Figure 5:**
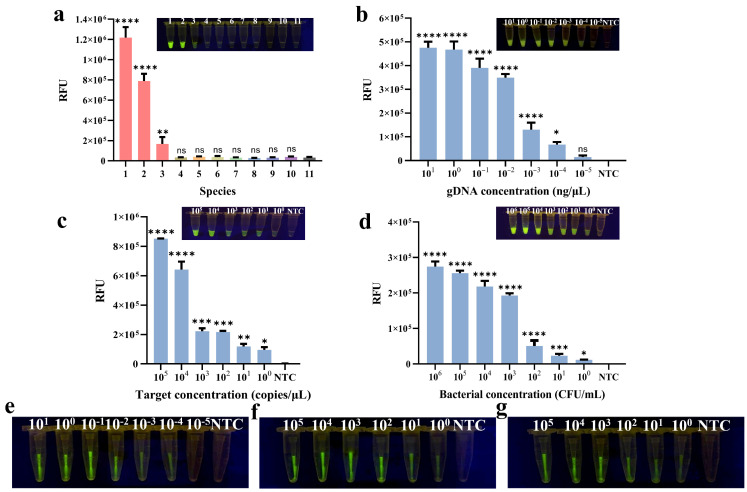
Sensitivity and specificity analysis of the two-step RPA-CRISPR/Cas12a method and PTIT method. (**a**) Specificity analysis of the two-step RPA-CRISPR/Cas12a method. Numbers from 1 to 11: *C. sakazakii*, *C. malonaticus*, *C. turicensis*, *E. coli*, *S. aureus*, *Salmonella*, *B. citreus*, *B. cereus*, *V. parahaemolyticus*, L. *monocytogenes*, no target control. Sensitivity of *ompA* in the two-step RPA-CRISPR/Cas12a method for (**b**) gDNA, (**c**) target DNA, and (**d**) pure bacterial culture. Sensitivity of the PTIT method was evaluated for (**e**) gDNA, (**f**) target DNA, and (**g**) pure bacterial culture by the naked eye. Error bars represent the mean ± standard deviation (SD) from three replicates. NTC represents no target control. One-way ANOVA test was used to compare all groups with no target control: * *p* < 0.05; ** *p* < 0.01; *** *p* < 0.001; **** *p* < 0.0001; ns, not significant.

The specificity of the methods was evaluated when the two-step method was established primarily. Several foodborne pathogens were tested, including three *Cronobacter* strains as well as *E. coli*, L. *monocytogenes*, *S. aureus*, *Salmonella*, *B. cereus*, *B. citreus*, and *V. parahaemolyticus*. The results demonstrated that only *C. sakazakii*, *C. malonaticus*, and *C. turicensis* could activate Cas12a cleavage activity, resulting in fluorescence signals (Figure 5a). Therefore, the method exhibited good specificity for the *Cronobacter* genus, and no cross-reactivity with other foodborne pathogens. Since the target fragment and all reagents are the same in the PTIT method, there is no need to verify the specificity of the PTIT method again.

The sensitivity of the two-step method was evaluated from three different levels: the concentrations of gDNA, pure bacterial culture, and target DNA. As shown in Figure 5b, visible fluorescence was observed and remained significantly different compared with NTC at a gDNA concentration of 10^−4^ ng/μL. For pure bacterial culture, the concentration threshold where obvious fluorescence was observed by the naked eye was 6 CFU/mL (Figure 5d). Additionally, the target DNA fragment was also subjected to a 10-fold serial dilution, providing a detection limit of 1.43 copies/μL (Figure 5c).

For comparison, the sensitivity of the PTIT method was reassessed using the same approach as the two-step method. As shown in Figure 5e,f, the fluorescence that remained visible to the naked eye was shown at concentrations as low as 10^−4^ ng/μL for gDNA, 6 CFU/mL for pure bacterial culture, and 1.43 copies/μL for the target DNA fragment. That means the sensitivity of the PTIT method was just as high as the two-step method (Figure 5g). At the same time, in order to measure the fluorescence intensity precisely, the pipette tip was cut short to be placed into the PCR tube. The endpoint fluorescence values were measured using Real-Time PCR to make more accurate judgements. The same detection limits were obtained by exact data analysis (Figure S2).

In recent years, rapid identification methods for *C. sakazakii* based on molecular biology, immunology, and biosensing were developed to meet the demand of the rapid detection of foodborne pathogens. For example, immunomagnetic beads have been used to detect and separate *C. sakazakii* in milk and europium chelate nanoparticles as signal probes for a fluorescence immunoassay. The entire detection process was completed within 2 h with a sensitivity of 73 CFU/mL [41]. Gao’s team combined improved propidium monoazide (PMAxx) with real-time recombinase polymerase amplification (qRPA) and Real-Time PCR to differentiate and quantify live and dead *C. sakazakii* cells with a sensitivity of 164 CFU/mL [42]. These methods ssignificantly improved detection performance, but they still had some defects, like requiring several hours, having relatively low sensitivity, relying on large equipment, or involving complex apparatus designs and skilled operators. In this study, the sensitivity of both the two-step method and the PTIT method reaches the highest level among all existing detection methods for *C. sakazakii* without the assistance of any large-scale equipment. Especially, the PTIT method overcomes the common aerosol contamination problem in isothermal nucleic acid detection.

### 3.7. Application of Methods in Food Samples

The practical applicability of the two methods was evaluated in samples of infant cow milk powder and infant goat milk powder. In this study, a series of gradient dilutions of pure bacterial cultures were artificially spiked into two types of milk powders, with final contamination concentrations ranging from 10^6^ CFU/mL to 10^0^ CFU/mL. Two different methods of DNA extraction from food samples, utilizing the commercial kit and rapid boiling, were tried at the same time.

**Figure 6:**
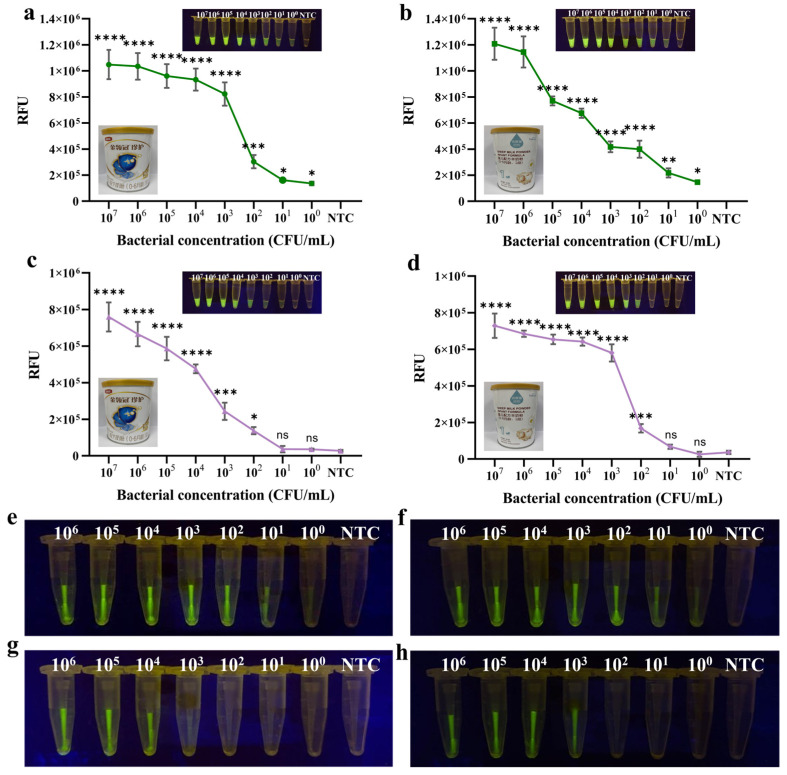
Evaluation of methods in artificially *C. sakazakii*-contaminated food samples. Commercial kits were used to extract gDNA from artificially contaminated (**a**) infant cow milk powder and (**b**) infant goat milk powder in order to assess the performance of the two-step RPA-CRISPR/Cas12a method for practical applications. The use of the rapid boiling method to extract gDNA from artificially contaminated (**c**) infant cow milk powder and (**d**) infant goat milk powder was employed to assess the performance of the two-step RPA-CRISPR/Cas12a method for practical applications. Results of the PTIT method for detecting artificially contaminated (**e**) infant cow milk powder and (**f**) infant goat milk powder samples (with gDNA extracted by the kit-based extraction method). Results of the PTIT method for detecting artificially contaminated (**g**) infant cow milk powder and (**h**) infant goat milk powder samples (with gDNA extracted by the rapid boiling extraction method). Error bars represent the mean ± standard deviation (SD) from three replicates. NTC represents no target control. One-way ANOVA test was used to compare all groups with no target control: * *p* < 0.05; ** *p* < 0.01; *** *p* < 0.001; **** *p* < 0.0001; ns, not significant.

For the two-step method, the detection limits for *C. sakazakii* were 4.65 CFU/mL in cow milk powder and 4.35 CFU/mL in goat milk powder, using the commercial DNA extraction kit. These values were nearly consistent with the detection limit for pure bacterial culture (Figure 6a,b). In contrast, when the samples were simply boiled for 10 min to extract DNA, the results seemed to be significantly impacted by the food matrix. The detection limits were increased to 4.65 × 10^2^ CFU/mL for the cow milk powder and 4.35 × 10^2^ CFU/mL for the goat milk powder, which were two orders of magnitude higher than the kit-based extraction method (Figure 6c,d).

The same assays were carried out for the PTIT method. The detection limits had no difference with that of the two-step method using the DNA extraciton kit (Figure 6e,f). However, the detection limits further increased to 4.65 × 10^3^ CFU/mL in cow milk powder and 4.35 × 10^3^ CFU/mL in goat milk powder (Figure 6g,h). The fluorescence intensity was also analyzed by Real-Time PCR to avoid visual misjudgment (Figure S3). It seems that the rapid boiling method had more impact on the PTIT method’s performance than the two-step method. The reason may be that not all of the RPA amplicons in the pipette tip could be thrown into and mixed with the Cas system in the outer tube immediately. Hence, the effect brought on by the impurity of the DNA template seems more apparent.

Obviously, the DNA purity obtained from the kit-based method was higher than the rapid boiling method. Therefore, the conclusions of the two methods in this study are based on the gDNA extracted by the kit-based extraction method, and the use of gDNA extracted by the rapid boiling method is not considered. However, in our previous study, we found that, in coconut water samples, the rapid boiling led to even better detection results than the kit [38]. In comparison, the milk powder samples contain a large amount of protein and lipids, which could not be removed by simple boiling, and thus lower the extracted DNA quality. Nonetheless, the rapid boiling method could reduce the loss of DNA during extraction when the sample matrix is simple.

### 3.8. Method Validation

To confirm the reliability of the established methods, the detection performance of the two-step RPA-CRISPR/Cas12a method and the PTIT method were compared with the Real-Time PCR method. The Ct values for pure bacterial cultures with concentrations ranging from 10^3^ CFU/mL to 10^0^ CFU/mL are shown in Figure S4. The Ct values of qPCR detection at these concentrations of pure bacterial cultures were 27, 30, 32, and 33, respectively. A Ct value of ≤35 was defined as a positive result for *C. sakazakii*. The results obtained from Real-Time PCR were consistent with the RPA-CRISPR/Cas12a and PTIT methods. This indicated that the methods developed in this study are reliable.

## 4. Discussion

This study proposed a novel tube-in-tube operation method based on physical separation, which has been applied to the detection of *C. sakazakii*. Compared with the conventional two-step RPA-CRISPR/Cas method, this method exhibits comparable sensitivity. Most importantly, during the process of establishing the PTIT method, all experiments were conducted in the same space without any occurrence of false-positive results. Similarly, compared with the existing one-pot methods, the sensitivity of the PTIT method has also reached the highest level among current methods. Additionally, compared with the physically isolated contamination-free one-pot methods that require customizing special tube-in-tube devices or designing centrifugal microfluidic chips, the PTIT method does not need additional device fabrication and can be operated using only the most common laboratory consumables, offering simplicity, convenience, and no extra costs. Similarly, in contrast to methods that utilize chemically modified crRNAs—such as those designed with suboptimal PAM sites or photo-controlled crRNAs—the PTIT method features extremely low costs and a high universality. However, during the operation process of the PTIT method, the back pressure when removing the pipette tip and the pressure when cutting the tip will cause the air bubble trapped at the tip of the pipette tip to shrink and shift forward in position. However, since aspirating the liquid via reverse pipetting and gently removing the pipette tip can reduce negative pressure, while controlling the cutting force can also alleviate the pressure, some air bubbles are still trapped in the pipette tip. This allows it to achieve the goal of isolating the two systems in the centrifuge tube containing the Cas system. Nevertheless, this does test the operational skills of laboratory operators and requires operation by professional or experienced laboratory personnel. Thus, the PTIT method is expected to promote the market application of CRISPR diagnostic technology, especially in grassroots laboratories, and provide an effective strategy to realize rapid, sensitive, and contamination-free diagnosis at the same time.

## 5. Conclusions

In this study, a specific fragment was screened out from the virulence gene *ompA* of *C. sakazakii*, leading to the first establishment of a visual detection platform based on RPA-CRISPR/Cas12a for *C. sakazakii*. To avoid the aerosol contamination that usually happened in the two-step procedure, a special one-pot method, the PTIT method, was developed by utilizing the capillary principle to physically isolate the RPA and CRISPR/Cas systems. This PTIT method can rapidly detect *C. sakazakii* with a detection limit of 10^0^ CFU/mL in both pure bacterial culture and food samples, without any enrichment. The sensitivity of the PTIT method is at the highest level among the existing methods, but is the least time-consuming. By changing the RPA primers and crRNA, people could utilize the PTIT method to detect other pathogens easily. Especially for researchers in laboratories where space is limited and poorly ventilated, this PTIT one-pot method can greatly simplify operational steps and control aerosol contamination effectively. It can be expected that one-pot methods will realize the practical application of the CRISPR/Cas detection system in grassroots laboratories and on-site rapid detection fields, featuring high sensitivity, accuracy, and operational convenience.

## Data Availability

The original contributions presented in the study are included in the article/Supplementary Materials. Further inquiries can be directed to the corresponding author.

## Supplementary Materials

The following supporting information can be downloaded at https://www.mdpi.com/article/10.3390/foods14173120/s1. Table S1: Six sets of primer sequences and sizes; Table S2: Sequences for RPA-CRISPR/Cas12a; Figure S1: Optimization of RPA amplification conditions; Figure S2: The sensitivity of the PTIT method by instrumental reading of the endpoint fluorescence values; Figure S3: The limit of the PTIT method for artificially contaminated food samples were measured by instrumental reading of the endpoint fluorescence values for two DNA extraction ways; Figure S4: Methods confirmation and comparison by Real-Time PCR.
