# Supplementary material for: A Simple, Rapid, and Contamination-Free Ultra-Sensitive Cronobacter sakazakii Visual Diagnostic Platform Based on RPA Combined with CRISPR/Cas12a
Source: Foods. 2025 Sep 6;14(17):3120. doi: 10.3390/foods14173120 (PMC12427865; doi:10.3390/foods14173120)
Supplement: Supplementary file 1 [file foods-14-03120-s001.zip › foods-3825007-supplementary.pdf]

Table S1. Six sets of primer sequences and sizes.

Gene amplified	Primers	Sequence of primers	Length of product/bp
F1	Forward	CAGGCCGCACCGAAAGATAACACCTGGTACG	222 bp
R1	Reverse	GAAAGCGCCGTTTACAGTGTCGCCTTTATAC	
F2	Forward	GTAGCGCAGGCCGCACCGAAAGATAACACCTG	238 bp
R2	Reverse	CCTGAGCTTTGAAAGCGCCGTTTACAGTGTCG	
F3	Forward	AGTTCCACGATACCGGCTTTATCCCTAACGACG	186 bp
R3	Reverse	GCTGTACGCCCTGAGCTTTGAAAGCGCCGTTTA	
F4	Forward	GTTGGTCCCAGTTCCACGATACCGGCTTTA	180 bp
R4	Reverse	CTTTGAAAGCGCCGTTTACAGTGTCGCCTT	
F5	Forward	GTTGGTCCCAGTTCCACGATACCGGCTTTA	267 bp
R5	Reverse	GCCATACCATGCCGCCCAGACGGGTGTATA	
F6	Forward	GCCGCACCGAAAGATAACACCTGGTACGCAGG	229 bp
R6	Reverse	CCTGAGCTTTGAAAGCGCCGTTTACAGTGTCG	

Table S2. Sequences for RPA-CRISPR/Cas12a.

Name	Sequence
Target DNA	GTTGGTCCCAGTTCCACGATACCGGCTTTATCCCTAACGACGGCCCGACTC<br>ACGAAAGCCAGCTGGGCGCAGGCGCGTTCGGTGGTTACCAGGTTAACCCG<br>TACGTTGGTTTCGAAATGGGCTACGACTGGCTGGGCCGCATGCCGTATAAA<br>GGCGACACTGTAAACGGCGCTTTCAAAGCTCAGGGCGTACAGCTGACCGC<br>TAAACTGGGTTACCCGGTAACCGACGACCTGGACGTATACACCCGTCTGG<br>GCGGCATGGTATGGC
Primer F	GTTGGTCCCAGTTCCACGATACCGGCTTTA
Primer R	GCCATACCATGCCGCCCAGACGGGTGTATA
crRNA	UAAUUUCUACUAAGUGUAGAUUCCCUAACGACGGCCCGACUC
FQ-ssDNA	FAM-TTTTTT-BHQ1

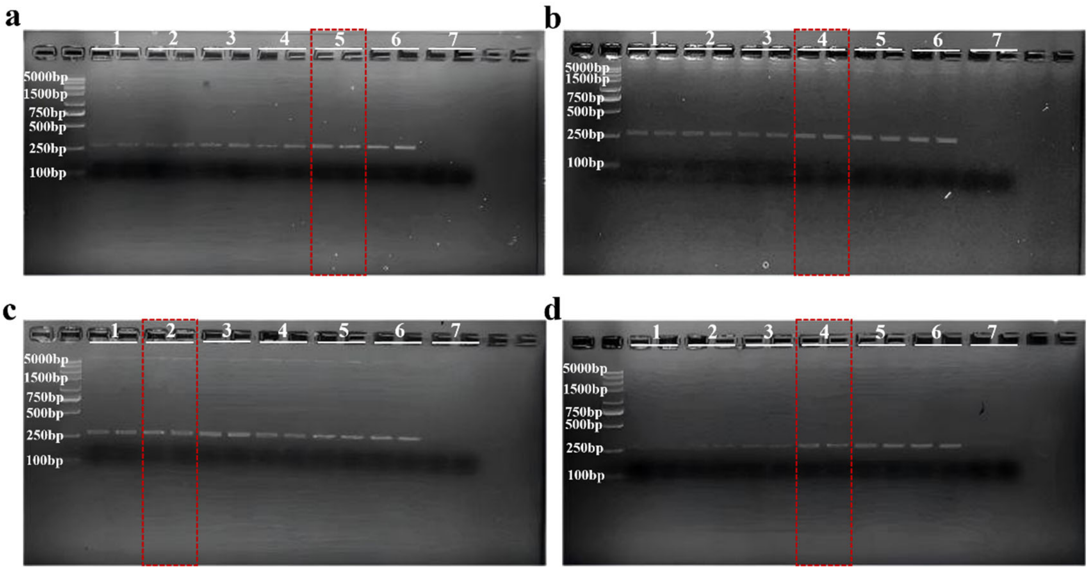

Figure S1. Optimization of RPA amplification conditions. **(a)** Single bands produced at different primer concentrations were visualized by 2% agarose gel electrophoresis. 1: 0.24 μM; 2: 0.32 μM; 3: 0.40 μM; 4: 0.48 μM; 5: 0.56 μM; 6: 0.64 μM; 7: no target control. **(b)** Single bands produced at different dNTPs concentrations were visualized by 2% agarose gel electrophoresis. 1: 0.80 mM; 2: 1.20mM; 3: 1.60mM; 4: 2.00 mM; 5: 2.40mM; 6: 2.80 mM; 7: no target control. **(c)** Single bands produced at different amplification temperatures were visualized by 2% agarose gel electrophoresis. 1: 36℃; 2: 37℃; 3: 38℃; 4: 39℃; 5: 40℃; 6: 41℃; 7: no target control. **(d)** Single bands produced at different amplification times were visualized by 2% agarose gel electrophoresis. 1: 10 min; 2: 15 min; 3: 20 min; 4: 25 min; 5: 30 min; 6: 35 min; 7: no target control.

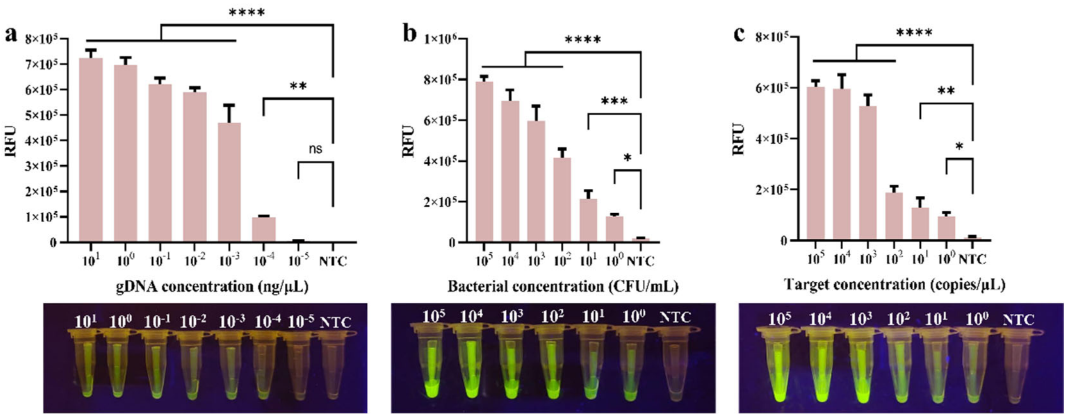

Figure S2. The sensitivity of the PTIT method by instrumental reading of the endpoint fluorescence values. **(a)** Sensitivity of the PTIT method at the level of gDNA. **(b)** Sensitivity of the PTIT method at the level of pure bacterial culture. **(c)** Sensitivity of the PTIT method at the target DNA level. Error bars represent the mean ± standard deviation (SD) from three replicates. NTC represent no target control One-way ANOVA test was used to compare all groups with no target control: *P <0.05; **P < 0.01; ***P <0.001; ****P <0.0001; ns, not significant.

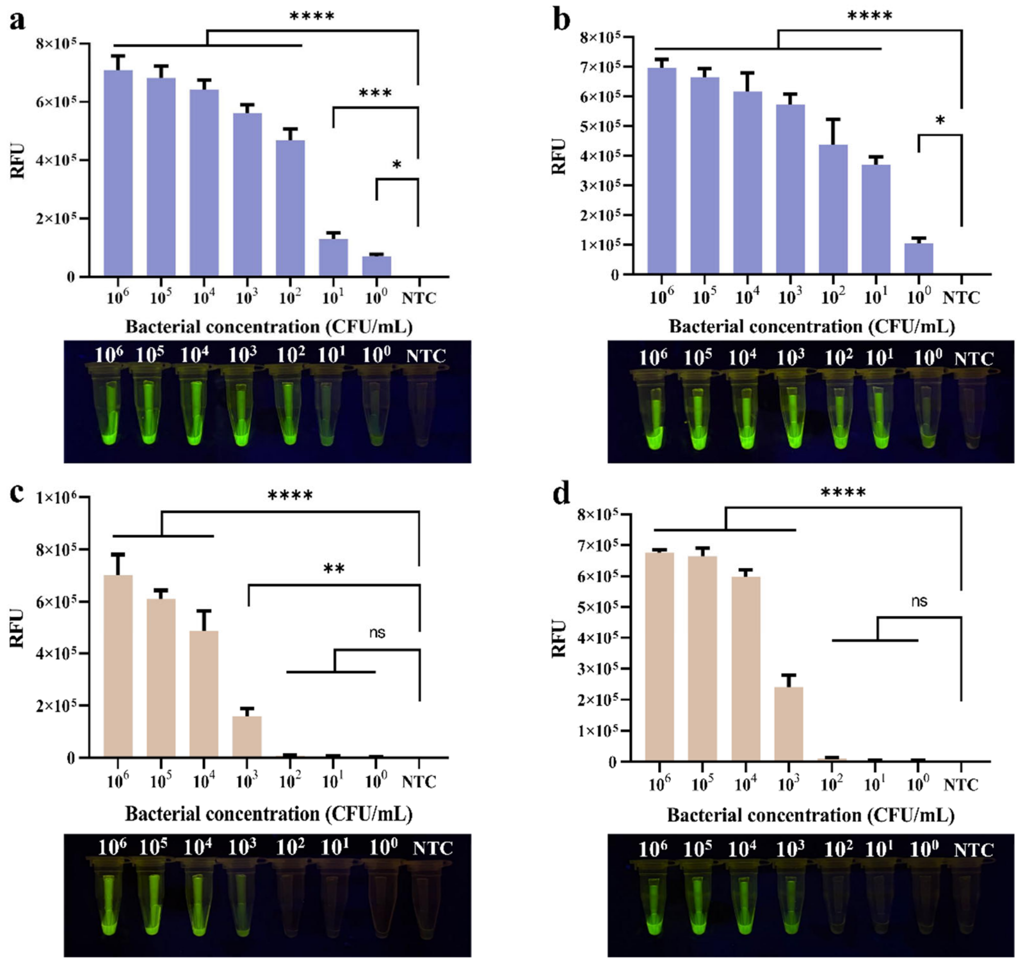

Figure S3. The limit of detection (LOD) of the PTIT method for artificially contaminated food samples was determined via instrumental reading of endpoint fluorescence values for the two DNA extraction methods. Commercial kits were used to extract gDNA from artificially contaminated (a) infant cow milk powder (b) and infant goat milk powder in order to obtain the PTIT method's LOD for practical applications. For the rapid boiling extraction method, LOD determination results of the PTIT method on artificially contaminated (c) infant cow milk powder and (d) infant goat milk powder samples are presented below. Error bars represent the mean ± standard deviation (SD) from three replicates. NTC represents no target control. One-way ANOVA test was used to compare all groups with no target control: *P <0.05; **P < 0.01; ***P <0.001; ****P <0.0001; ns, not significant.

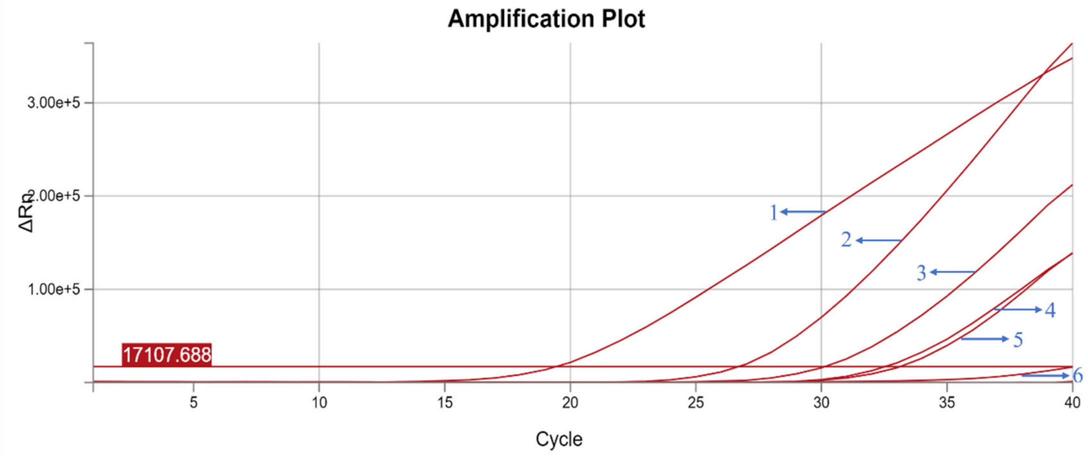

Figure S4. Methods confirmation and comparison by qPCR. Amplification curves at different concentrations of pure bacterial culture. 1: positive control; 2-5: 10^3 CFU/mL, 10^2 CFU/mL, 10^1 CFU/mL, 10^0 CFU/mL; 6: no target control.
